# Monitoring the Response of Cyclin-Dependent Kinase 4/6 Inhibitors with Mean Corpuscular Volume

**DOI:** 10.3390/curroncol31100424

**Published:** 2024-09-24

**Authors:** Bediz Kurt İnci, Pınar Kubilay Tolunay, Şura Öztekin, Ergin Aydemir, İrem Öner, Öztürk Ateş, Cengiz Karaçin

**Affiliations:** Dr. Abdurrahman Yurtaslan Ankara Oncology Hospital, Ankara 06560, Türkiye; pinar_kubilay@hotmail.com (P.K.T.); sura.oztekin@hotmail.com (Ş.Ö.); draydemirergin@gmail.com (E.A.); dr_iremoner@hotmail.com (İ.Ö.); dr.ozturkates@gmail.com (Ö.A.); cengizkaracin@yahoo.com (C.K.)

**Keywords:** mean corpuscular volume, CDK4/6, palbociclib, ribociclib

## Abstract

Background: Currently, the combination of cyclin-dependent kinase 4/6 (CDK4/6) inhibitors and endocrine therapy is a first-line treatment for hormone-receptor-positive and HER2-negative metastatic breast cancer. This study aimed to assess the impact of changes in Mean Corpuscular Volume (MCV) on predicting responses to treatment and survival in patients with hormone-receptor-positive, HER2-negative metastatic breast cancer receiving CDK4/6 inhibitors and endocrine therapy. Methods: Retrospectively, data on hemoglobin levels, MCV, B12, folate levels, and survival times were collected from 275 patients. Patients were categorized into two groups based on the degree of MCV change (delta MCV ≤ 10 vs. >10). Kaplan–Meier survival analysis was performed, with significance set at *p* < 0.05. Results: The average age of the patients was 56.1 ± 12.1 years. In total, 72.7% received CDK4/6 inhibitors as first-line treatment, while 27.3% received them as second-line treatment. Before CDK4/6 inhibitor use, the median MCV level was 87.7 fL (IQR: 83–91), which increased to 98 fL (IQR: 92–103) after treatment (*p* < 0.001). ECOG performance score, CDK4/6 inhibitor treatment line, type of endocrine therapy, and MCV change were identified as independent predictors of progression-free survival in the Cox regression model. The median progression-free survival for the entire group was 28 months. Patients with MCV delta > 10 had a median progression-free survival of 33 months, compared to 23 months for those with MCV delta ≤ 10 (*p* = 0.029). There was no significant difference in median overall survival times between the two groups (*p* = 0.158). Conclusion: This study highlights that patients with MCV delta > 10 had longer median progression-free survival compared to those with MCV delta ≤ 10.

## 1. Introduction

The combination of cyclin-dependent kinase 4/6 (CDK4/6) inhibitors and endocrine therapy is currently a first-line treatment option for hormone-receptor-positive (HR+) and HER2-negative (HER2−) metastatic breast cancer. CDK4/6 inhibitors have shown effectiveness in the first-line treatment of metastatic breast cancer in postmenopausal women through PALOMA-2, MONALEESA-2, and MONARCH-3 trials, and in premenopausal women through the MONALEESA-7 trial [1,2,3,4]. Specifically, palbociclib, ribociclib, and abemaciclib, the three available CDK 4/6 inhibitors, in combination with standard endocrine therapy have shown improvement in progression-free survival in phase III trials and have been approved for use in both first and later lines of therapy in women with HR+HER2− mBC, regardless of menopausal status, age, endocrine sensitivity, and type of metastasis.

However, patients with HR+HER2− mBC can develop resistance to CDK4/6 inhibitor-based combinations, with a percentage displaying rapid progression [5]. With these treatment options included in guidelines, studies are now focusing on parameters that predict treatment response to identify which patients will benefit the most in clinical practice [6]. Molecular markers that are believed to cause endocrine resistance and influence the response to CDK4/6 inhibitors are receiving significant attention, though biomarkers of intrinsic and acquired resistance to CDK4/6is therapy are currently lacking in clinical practice [7].

Efforts are being made to find cost-effective, easily applicable, and quickly accessible methods to predict treatment response in clinical practice and deliver the right treatment to the right patient. Parameters routinely assessed before treatment, such as modified Scarff–Bloom–Richardson scores, ER, HER2, PR immunohistochemistry, NOLUS score, and inflammatory scores, are still being investigated for their predictive value in treatment responses [8,9,10].

Mean Corpuscular Volume (MCV) is a method that has shown promise in predicting the response to CDK4/6 inhibitors [11,12]. Erythroblasts contain Cdk4 and Cdk6, while mature erythrocytes lack Cdk4, with their Cdk6 being partly associated with the cytoskeleton [13]. Inhibiting CDK6 leads to the disruption of the mature erythrocyte cytoskeleton, potentially explaining the macrocytosis associated with CDK4/6 inhibition. Few studies in the literature have evaluated the predictive value of MCV changes for treatment response, all of which have been conducted in limited patient groups. This study aims to assess the impact of MCV changes in predicting treatment response in HR+HER2− mBC patients using CDK4/6 inhibitors and endocrine therapy as a first- or second-line treatment option.

## 2. Materials and Methods

Between January 2019 and January 2023, a total of 450 patients with HR+/HER2− mBC treated with a CDK4/6i+ET were retrospectively screened at Dr. Abdurrahman Yurtaslan Ankara Oncology Hospital. Ethical approval for the study was obtained from the Clinical Research Ethics Committee of Dr. Abdurrahman Yurtaslan Ankara Oncology Hospital dated 14 September 2023 and numbered 2023/09-77. Data on initial and third-month Hb (hemoglobin) and MCV (mean corpuscular volume) values and initial B12 and folate levels were obtained from 390 patients. A total of 115 patients with an initial MCV value of >100 fL and low B12 and folate levels were excluded from the study (in our patients, we accepted the lower limit for vitamin B12 as 200 picograms per milliliter (pg/mL) and for folic acid as 3 nanograms per milliliter (ng/mL)). The study included only female patients who received CDK4/6i plus ET as first- or second-line treatment and were able to continue treatment for at least three months. In the premenopausal patient group included in our study, ovarian suppression was achieved simultaneously with goserelin treatment in combination with CDK4/6i plus ET.

The difference (increase, decrease, or remain constant) between the patients’ initial and third-month MCV values was calculated, and referred to as the MCV delta. The median value was used as the cut-off for data distribution since a significant *p*-value of less than 0.001 was detected according to the Kolmogorov–Smirnov test. The median MCV change was calculated as 10.0 fL (min–max: −12.8–24.0) (IQR: 7–14). Patients were divided into two groups based on their MCV delta, categorized as low and high according to the median value.

Disease control rate (DCR) was defined as the sum of complete remission (CR), partial remission (PR), and stable disease (SD) rates. The overall response rate (ORR) was defined as the sum of complete remission (CR) and partial remission (PR) rates.

### Statistical Analysis

A Wilcoxon test was used to analyze repeated nonparametric measurements. The difference in numerical continuous variables between the two groups was evaluated using Student’s *t*-test. The Chi-squared test was used to describe the relationship between two separate categorical groups. Kaplan–Meier survival analysis and Cox regression analysis were also conducted for multivariate survival analysis.

## 3. Results

The final analysis included a total of 275 patients. The mean age of the patients was 56.1 ± 12.1 years, and the median follow-up duration was 35 (IQR: 31.5–38.5) months. The median hemoglobin (Hb) value was 12.9 g/dL (IQR: 11.9–13.7 g/dL) before CDKi and 11.9 g/dL (IQR: 10.9–12.8 g/dL) after treatment (*p* < 0.001). The MCV level was found to have a median of 87.7 fL (IQR: 83–91) before CDK4/6i use and a 98 fL (IQR: 92–103) after CDK4/6i use (*p* < 0.001). The median value of MCV delta was calculated as 10.0 fL (min–max: −12.8–24.0) (IQR: 7–14). Patients with MCV delta ≤ 10 fL were classified as low MCV delta, and those with MCV delta >10 fL were classified as high MCV delta.

When demographic data were examined, no significant difference was found between the low and high MCV delta groups in terms of ECOG performance status, comorbidity, metastatic status, metastatic site, treatment line, CDK4/6 inhibitor choice, and type of ET (*p* = 0.514, *p* = 0.568, *p* = 0.238, *p* = 0.278, *p* = 0.902, *p* = 0.761, *p* = 0.732, respectively) (Table 1). Significant differences were found between the groups regarding age and menopausal status (*p* < 0.001, *p* < 0.001, respectively, Table 1).

In univariate analysis, factors associated with low PFS were evaluated as ECOG performance status, treatment line, CDK4/6 inhibitor choice, and type of ET and MCV delta (Table 2). These factors were included in the multivariate analysis, and the ECOG performance score, treatment line, type of ET, and MCV delta affected PFS significantly (Table 2).

**Figure 1:**
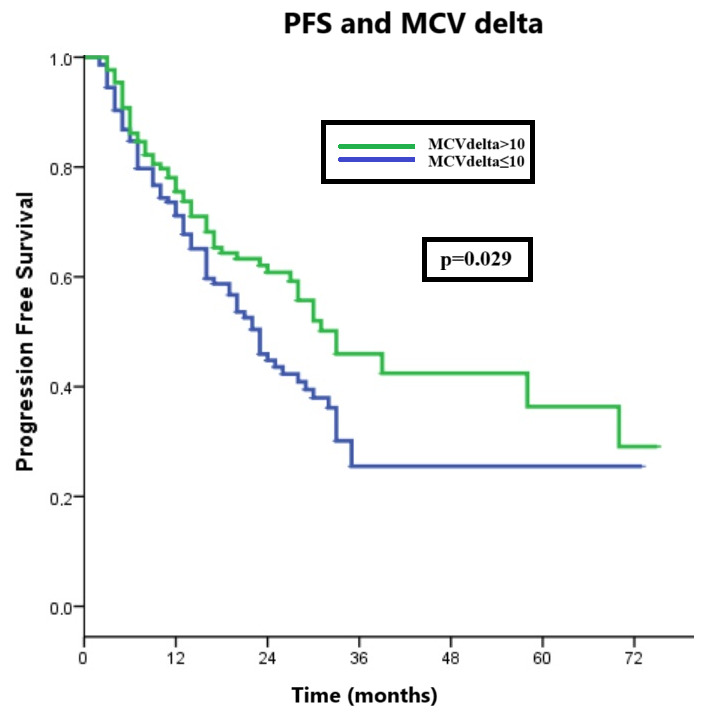
Progression-free survival and MCV delta.

**Figure 2:**
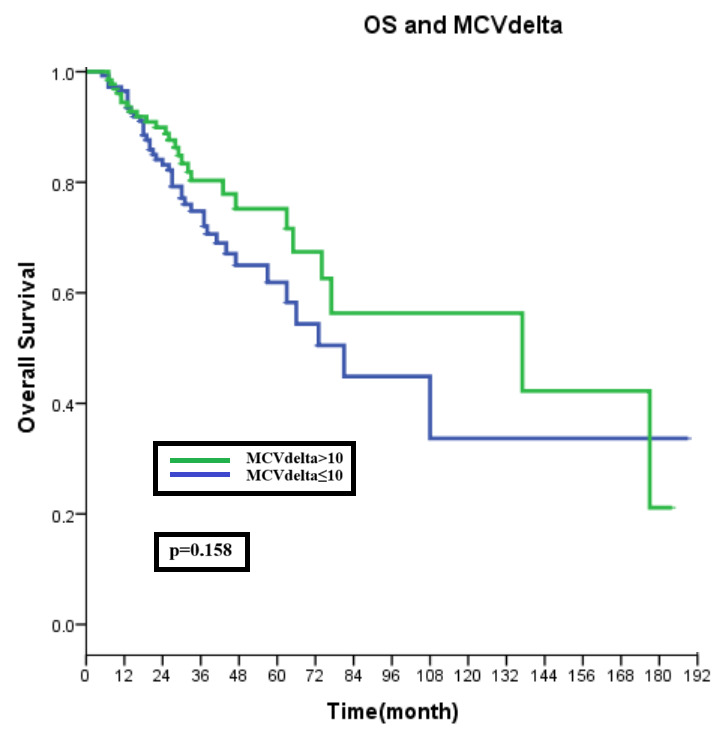
Overall survival and MCV delta.

In the overall group, the median PFS was 28 (95% CI: 23.3–32.7) months. When the PFS times of the low and high MCV delta patient groups were evaluated, while the median PFS time of low-MCV-delta patients was 23 (95% CI: 19.1–26.9) months, the median PFS time in the high patient group was 33 (95% CI: 23.6–42.4) months (*p* = 0.029, Figure 1). The groups had no significant difference regarding median OS times (*p* = 0.158, Figure 2).

In the third-month disease response evaluations, in the overall group, 0.7% (2) CR, 19.3% (53) PR, 64.7% (178) SD, and 15.3% (42) PD were observed. DCR was 84.7%, and ORR was 20%. There was no difference between the MCV delta groups regarding third-month disease radiological response evaluation, DCR, and ORR (*p* = 0.425, *p* = 0.103, *p* = 1.00, respectively).

## 4. Discussion

Our study focused on defining the predictive impact of MCV elevation associated with CDK4/6i+ET use on survival. The first definitive study on CDK4/6i+ET causing macrocytosis was published in 2017 [14]. In this study by Haque et al., 32 patients on palbociclib for more than three months were analyzed, and it was found that median MCV increased significantly after treatment compared to baseline. Sixteen patients developed macrocytosis, and progression-free survival (PFS) was significantly higher in this group. However, this difference in PFS was not seen when the change in MCV was analyzed as a time-dependent variable. In the study by Kamboj et al., all patients (n = 6) had an increase in MCV within 4–6 weeks after initiation of the CDK4/6 inhibitor, and the time for MCV to rise above 100 ranged from 3 to 7 months. In 2018, Anampa et al. evaluated 34 patients on palbociclib therapy, and 16 (47%) of them developed macrocytosis. It was shown that median MCV values increased significantly after four cycles of drug use compared to baseline [11,15]. In our study, we aimed to evaluate the MCV detected after three months of treatment by evaluating the definitions of the time of MCV elevation in these three studies. In our study, in 48% of patients (n = 130), the MCV at the third month increased by more than ten units compared to baseline. Could this increase be a parameter to predict how long patients will benefit from CDK4/6i+ET treatment? That becomes the question of our study.

Similar to the studies in the literature, our study detected a significant increase in MCV with CDK4/6i+ET compared to baseline [11,12,14,15]. When the patient groups were separated according to the concept of delta MCV developed based on the median MCV value, as in the study by Anampa et al., differences were observed only in age and menopausal status. The parameters shown to affect PFS via univariate analysis were ECOG performance status, treatment line, choice of CDK4/6 inhibitor, and type of endocrine therapy. Only the choice of CDK4/6 inhibitor was effective on PFS, contrary to the literature, while the other parameters were similar to the literature [8]. Comparative retrospective evaluations have been conducted to address whether there are differences in efficacy due to small differences in the mechanisms of action of CDK4/6 inhibitors (palbociclib and ribociclib used in our study), as described in the literature. These studies have not detected any differences in their effects on clinical outcomes such as PFS [16]. It was considered that one of the reasons for the effective impact of CDK4/6 inhibitor selection on PFS might be the preference for palbociclib in elderly patients at our center due to its side effect profile. In addition to the mentioned parameters, MCV delta was also significant for PFS in univariate analyses. When all significant parameters were included in the multivariate analysis, the significance of MCV delta on PFS was maintained. Our study is significant in this regard as it addresses the evaluation of MCV in multivariate analysis, which has yet to be discussed in most studies focusing on the impact of MCV increase on PFS in the literature [10]. Our study found similar overall response rate (ORR) and disease control rate (DCR) rates in the first three months among MCV delta groups. This is the first study to evaluate the relationship between radiological treatment response and increased MCV from CDK4/6 inhibitor+ET.

In the first-line metastatic setting, the median progression-free survival for the three currently approved CDK4/6 inhibitors, palbociclib, ribociclib, and abemaciclib, with aromatase inhibitors is greater than two years (palbociclib 27.6 months; ribociclib 25.3 months; and abemaciclib 28.18 months) [2,17,18]. However, after a course of endocrine therapy in the second line, this period decreases to 9.5 months [19]. Similar to the literature, our study observed a median PFS of 28 months in the overall group (first-line and second-line treatment). When subgroups were examined, our study demonstrated a ten-month difference in which PFS was more advantageous in the high MCVdelta group. However, no significant difference was found in overall survival between the groups. Therefore, other factors, such as treatments received in subsequent lines, may have an impact. Nevertheless, this aspect was not considered within the primary objectives of our study.

Very few studies in the literature evaluate the relationship between MCV increase and PFS. One of these studies, conducted by Anampa et al., showed that 24 patients with an increase in MCV value of more than 10 fL could receive treatment longer than others (10 months vs. 5 months). Additionally, the median PFS was significantly longer in patients with delta MCV > 10 fL than those with MCV < 10 fL. However, when delta MCV of 10 fL or more was included as a time-dependent variable, this difference was not statistically significant [11].

In another study that evaluated this, all patients with a positive MCV shift after treatment were compared with those without, without determining a cut-off value. The study found a higher PFS in the group with a positive MCV shift [10]. This study used multivariate analysis similar to ours, and contrary to the results of other studies, it was shown that PFS was prolonged in patients with increased MCV even in multivariate analysis. Our study supports these data, and the effect of MCV delta on PFS continues in multivariate analyses.

Myelodysplastic syndromes (MDS) are a group of disorders characterized by dysplastic blood cells across various lineages in the bone marrow, and one of the common issues that this condition can cause in blood parameters is an increase in MCV. These syndromes are most commonly primary MDS, which has an unknown cause, and secondary MDS, which has a well-defined etiology often linked to prior oncological treatments. In our dataset, there were no additional findings suggestive of myelodysplastic syndrome (MDS) such as pancytopenia, so no patients required bone marrow biopsies. However, the literature does indicate the development of MDS in some cases, especially with long-term follow-up [20]. Therefore, future studies with extended follow-up and collaboration with hematology will be valuable.

This study has the highest number of patients evaluating the positive relationship between the increase in MCV resulting from CDK4/6i+ET and PFS in HR+HER2− mBC patients. Additionally, in the existing studies on this topic in the literature, palbociclib has been predominantly used as the CDK4/6 inhibitor, while our study includes both palbociclib and ribociclib patients, which also stands out as another parameter increasing the reliability of the study. The presence of an age-related change in MCV, while not substantial, has been shown to be statistically significant in some studies [21]. Therefore, one of the limitations of our study is that the mean ages of the MCV delta groups differ. Another limitation is the presence of a parameter related to menopausal status that was found to be different among MCV delta groups, and the unknown effect of menopausal status on MCV values with age. The effect of MCV delta on PFS did not translate to the OS analysis, as its impact is multi-parametric. Data on subsequent treatments and mutational analyses for patients who progressed under CDK4/6 inhibitors plus endocrine therapy were not included in our study, which is a limitation. Commenting on this issue would be more accurate in a study where these fundamental parameters are incorporated into multivariate analyses to assess their effect on OS.

In conclusion, our study demonstrates that the increase in MCV seen after treatment compared to baseline in HR+HER2− mBC patients treated with CDK4/6i+ET positively impacts PFS. This indicates that regularly analyzing blood counts can help assess side effects during the follow-up of this treatment, providing a simple and cost-effective way to predict patient progression-free survival.

## Figures and Tables

**Table 1:** Demographic data of first-line CDK4/6 inhibitors per MCV delta subgroup.

Variable	Total	MCV ≤ Delta10fl	MCV > Delta10fl	*p*-Value
n	275	145	130	
Age (mean ± std)	56.1 ± 12.1	53.1 ± 12.3	59.5 ± 10.9	<0.001 *
ECOG (n%)				
0–1	262 (95.3)	137 (94.5)	125 (96.2)	0.514
>1	13 (4.7)	8 (5.5)	5 (3.8)	
Comorbidity				
Yes	143 (52)	72 (49.7)	71 (54.6)	0.568
No	132 (48)	73 (51.3)	59 (45.4)	
Menopausal status (n%)				
Post-menopausal	189 (68.7)	83 (57.2)	106 (81.5)	
Pre-menopausal	86 (31.3)	62 (42.8)	24 (18.5)	<0.001 *
Metastatic status (n%)				
De novo	123 (44.7)	60 (41.4)	63 (48.5)	
Recurrent	152 (55.3)	85 (58.6)	67 (51.5)	0.238
Metastatic site				
Non-visceral	170 (61.8)	94 (64.8)	76 (58.5)	0.278
Visceral	105 (38.2)	51 (35.2)	54 (41.5)	
CDK4/6 treatment line				
First-line	200 (72.7)	105 (72.4)	95 (73.1)	
Second-line	75 (27.3)	40 (27.6)	35 (26.9)	0.902
CDK4/6 Treatment option				
Ribociclib	173 (62.9)	90 (62.1)	83 (63.8)	
Palbociclib	102 (37.1)	55 (37.9)	47 (36.2)	0.761
CDK4/6 combination (n%)				
AI	194 (70.5)	101 (69.7)	93 (71.5)	
Fluvestrant	81 (29.5)	44 (30.3)	37 (28.5)	0.732

* Significant.

**Table 2:** Univariate and multivariate analyses to determine predictive factor for PFS.

Variables	Univariate Analyses			Multivariate Analyses		
	HR	CI 95%	*p* Value	HR	CI 95%	*p* Value
Age	1	0.99–1.01	0.592			
ECOG	2.48	1.30–4.75	0.006 *	2.97	1.53–5.74	0.01 *
0–1						
>1						
Comorbidity	0.81	0.58–1.14	0.229			
Yes						
No						
Unknown						
Menopausal status	1.08	0.75–1.55	0.683			
Post-menopausal						
Pre-menopausal						
Metastatic status	1.17	0.83–1.65	0.361			
De novo						
Recurrent						
Metastatic site	0.92	0.78–1.10	0.369			
Non-visceral						
Visceral						
CDK4/6 treatment line	1.88	1.32–2.66	0.001 *	1.75	1.19–2.55	0.04 *
First-line						
Second-line						
CDK4/6 Treatment option	1.41	1–1.98	0.050	1.19	0.84–1.69	0.329
Ribociclib						
Palbociclib						
CDK4/6 combination	1.76	1.23–2.53	0.002 *	1.52	1.04–2.23	0.032 *
AI						
Fluvestrant						
MCV delta (fL)	0.69	0.49–0.97	0.031 *	0.66	0.46–0.93	0.017 *
<10						
≥10						

* Significant.

## Data Availability

Due to patient rights and confidentiality regulations in our country, data sharing cannot be conducted directly, but upon request, data can be sent after consultation with the authors and the ethics committee.
